# Mucosal fluid evaporation is not the method of heat dissipation from fourth-degree laryngopharyngeal burns

**DOI:** 10.1038/srep28772

**Published:** 2016-06-28

**Authors:** Jiang-bo Wan, Guo-an Zhang, Yu-xuan Qiu, Chun-quan Wen, Tai-ran Fu

**Affiliations:** 1 Department of Burns Surgery, Peking University Fourth School of Clinical Medicine, No. 31, Xinjiekou East Street, Xicheng District, Beijing 100035, PR China; 2 Key Laboratory for Thermal Science and Power Engineering of Ministry of Education, Department of Thermal Engineering, Tsinghua University, No. 1, Tsinghua Park, Haidian District, Beijing 100084, PR China

## Abstract

This study was designed to explore whether mucosal fluid evaporation represents a method of heat dissipation from thermal air inhalation injury and to assess laryngopharyngeal tissue damage according to heat quantity changes of dry air and vapour. Fifteen adult male beagles were divided into five groups to inhale heated air or vapour for 10 min as follows: control group (ordinary air), group I (91–110 °C heated air), group II (148–175 °C heated air), group III (209–227 °C heated air), and group IV (96 °C saturated vapour). The heat quantity changes of the dry air and vapour were calculated via thermodynamic formulas. The macroscopic and histological features of the laryngopharynxes were examined and assessed by various tissue damage grading systems. Group IV exhibited the most serious laryngopharyngeal damage, including cilia exfoliation, submucosal thrombosis, glandular atrophy, and chondrocyte degeneration, which is indicative of fourth-degree injury. The quality, heat quantity, and proportional reduction of heat quantity of vapour in group IV were all higher than those in the other groups. Furthermore, we found that mucosal fluid evaporation is not the method of heat dissipation from thermal air inhalation injury used by the airways. Laryngopharyngeal tissue damage depends chiefly on the heat quantity of vapour in the air.

Inhalation injury is a major cause of morbidity and mortality in burn patients. The presence of inhalation injury, the total burn surface area, and the patient’s age are the three most significant predictors of death after thermal injury. The incidence of inhalation injury in burn patients who require hospitalization ranges from 20% to 30%. Up to 30% mortality has been reported among those with inhalation injury, mainly as a result of smoke inhalation^1^. However, the physical thermal air is known to more often cause upper airway injury than lower airway or pulmonary parenchyma injury.

Mucosal fluid evaporation has been reported as one possible self-protective mechanism from thermal injury of the airway^2^,^3^,^4^,^5^. Mucosal fluid protects the airway by absorbing and evaporating heat from the inhaled thermal air. However, preliminary experiments from our group have shown that mucosal fluid evaporation is not the main process occurring in the airway and not the method used for heat dissipation of thermal air (data not shown).

In recent decades, most studies on inhalation injury have focused largely on injury to the trachea and lung tissues due to thermal/chemical factors, such as smoke inhalation, and most of these studies have examined damage at the cellular and molecular level^6^,^7^,^8^,^9^,^10^. However, only a few studies have focused on damage to the upper airway, in particular the laryngopharynx. Hence, although the clinical manifestations of the laryngopharynx are commonly used to evaluate the severity of inhalation injury and for planning subsequent treatments^11^,^12^, the extent of damage to the laryngopharynx after inhalation injury remain largely unknown.

To fill this gap in knowledge, we designed the present study based on thermodynamic methods to explore the mass changes of vapour and the changes in heat quantity of dry air and vapour during the process of thermal air inhalation. We also explored the relationships between these changes and laryngopharyngeal tissue damage as a means to better recognize and understand inhalation injury.

## Methods

### Materials

A custom-made electrical air heating tube (Fig. S6, designed with CATIA v5 software, Dassault, France) comprising a spiral ceramic stick (length, 14 cm; diameter, 1 cm), electrothermic wire (733 W), corundum tube (length, 20 cm; inner diameter, 2.5 cm; external diameter, 3.1 cm), digital temperature indicator (XMT-7; Beijing Kunlun Hengye Electronic Technology Co. Ltd., China), alternating current contactor (AC contactor; CJX2-1210; Delixi Electric Co. Ltd., China), and thermal resistance probe (tp100; length, 3 cm; diameter, 0.3 cm; Beijing Kunlun Hengye Electronic Technology Co. Ltd.), was used in this study. This custom-made tube was designed such that when the temperature detected by the probe is higher than the set temperature, the digital temperature indicator cuts the circuit between the AC contactor and the electrothermic wire, and when the detected temperature is lower than the set temperature, the digital temperature indicator reconnects the AC contactor to the circuit of the electrothermic wire. Using this device, airflow can be heated to and maintained around the target temperature.

Moreover, a custom-made vapour generator (Fig. S7, designed with CATIA v5 software, Dassault, France) comprising a cubic ceramic container (length, 20 cm; width, 20 cm; height, 40 cm; wall thickness, 1 cm), built-in heating stick (600 W), built-in stainless intake tube (diameter, 1 cm), and a T-type thermotolerant rubber tube (inner diameter of horizontal axis, 4 cm; inner diameter of vertical axis, 2.5 cm), was used. The heating stick heated the deionized water until boiling and kept it heated throughout the experimental periods; as a result, the cubic container was filled with saturated vapour outflowing through the T-type rubber tube.

The temperature and humidity detecting instruments mainly included sensors (HC2-IC102 and HC2-C04; ROTRONIC AG, Switzerland) and related software (HW4-E; ROTRONIC AG). Other materials used in this study included an endoscope (ME-818W5; Beijing Yingmei Yihe Electronic Technology Co. Ltd.), digital camera (EX-ZS35, CASIO, Japan), microscope (CX31, Olympus, Japan), surgical instruments, and other necessary experimental instruments and chemical reagents.

### Animal grouping

This study was reviewed and approved by the Animal Ethics Committee and animal care research committee of Beijing Jishuitan Hospital (Permit Number: 201411-01). All experiments were performed in accordance with the institutional animal care research protocols. Fifteen healthy male adult beagles, weighing 10.75 ± 0.34 kg were supplied by Beijing Vital River Laboratory Animal Technology Co. Ltd., and randomly allocated into five groups (three in each group): control group (inhaled ordinary air), group I (inhaled heated air at 91–110 °C), group II (inhaled heated air at 148–175 °C), group III (inhaled heated air at 209–227 °C), and group IV (inhaled saturated vapour at 96 °C).

### Experimental procedures

After 24 h of fasting, the dogs were anesthetized with 2% pentobarbital sodium (1.5 ml/kg) and sumianxin II (0.15 ml/kg) along with normal saline via an intravenous injection into the cutaneous vein of the forelimb. Atropine sulphate (0.05 mg/kg) was subcutaneously injected prior to the anaesthesia. The dogs were placed in the supine position and the upper jaw was immobilized to keep the head from falling backward. Respiratory rate, blood oxygen saturation level, and pulse were continuously monitored and recorded every 2 min during the experiment. Two rubber plugs were inserted into the nostrils to compel the dogs to breathe through the oral cavity. The tongues were pulled out to avoid glossoptosis. Hair was removed in a 20 cm × 30 cm area at the anterior and lateral region of the neck. After standard disinfection and draping, we cut the skin open to expose the cricoid cartilage. Temperature and humidity sensors were inserted into the trachea at the cricoid cartilage to detect the temperature and relative humidity (RH), respectively, of the inhaled air. The end of the heating or rubber tube, which was located deep in the oral cavity, was approximately 5 cm from the epiglottis; therefore, breathing had to be performed via the tube channel. The time of heated air or vapour inhalation was 10 min. All experiments were conducted in room temperature of 26.28 ± 1.22 °C and air humidity of 54.24 ± 5.68%.

After the experiment, we immediately used an endoscope to observe any mucosal injury of the laryngopharyngeal cavity. Subsequently, the dogs were euthanized. Tissue samples of the epiglottis, thyroid cartilage, and cricoid cartilage were collected (thickness of 3 μm) and fixed in 4% paraformaldehyde, decalcified, embedded in paraffin, and sectioned for haematoxylin and eosin staining. The macroscopic and histological features of the laryngopharynx in the different groups were assessed by tissue damage assessing systems (Tables 1 and 2).

### Formula derivations

#### Formulas used for calculations of the heat quantity of dry air and vapour

Firstly, we should note that the thermodynamic concept of air (also called moist air) includes dry air and vapour, irrespective of whether the air is ordinary air, thermal air, or saturated vapour. The mass of the dry air between the heating tube (or rubber tube) and the airway is equal only at one cycle of respiration. Here, we used the data from the heating or rubber tube (not the airway) for the calculations of dry air mass^13^,^14^,^15^, as follows:


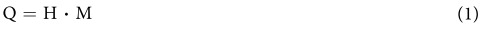


where Q is the quantity of heat of dry air (or vapour); H is the enthalpy of dry air (or vapour), which is defined as the total heat quantity of dry air (or vapour) relative to a unit mass of dry air (or vapour) in kJ/kg; and M is the mass of dry air or vapour.


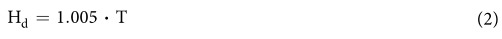



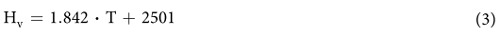


where H_d_ is the enthalpy of the dry air, H_v_ is the enthalpy of the vapour, and T is the temperature of the moist air. The constants 1.005 and 1.842 represent the isobaric-specific heat of dry air and vapour, respectively, which are defined as the specific enthalpy (i.e., the partial derivative of the temperature under invariable pressure) in kJ/kg∙K, and 2,501 is the value of the enthalpy of vapour at 0 °C. Using equations (2) and (3), the values were benchmarked against those at 0 °C.


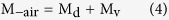



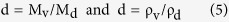


From formulas (4) and (5), we obtain formulas (6) and (7):


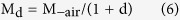



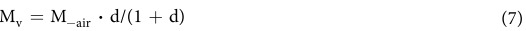


where M_−air_ is the mass of moist air; M_d_ is the mass of dry air; M_v_ is the mass of vapour; d is the specific humidity, which is defined as the mass of vapour coexisting with a unit mass of dry air in kg/kg (vapour/dry air); ρ_d_ is the density of dry air; and ρ_v_ is the density of vapour.


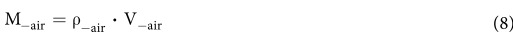



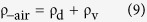



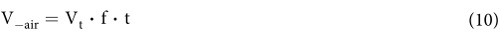


From formulas (5) and (8) to (10), we transformed formulas (6) and (7) into formulas (11) and (12), as follows:


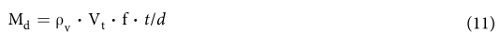



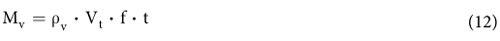


where ρ_−air_ is the density of moist air; V_−air_ is the volume of moist air; V_t_ is the tidal volume of the dog (300 ml)^16^; f is the respiratory rate; and t is the time of heated air inhalation.

#### Calculation of the heat quantity changes of dry air and vapour between the heating tube or rubber tube and airway


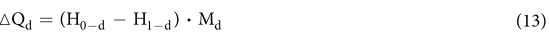



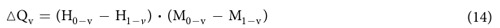


where △Q_d_ and △Q_v_ are the heat quantity changes of dry air and vapour, respectively; H_0−d_ and H_0−v_ are the enthalpies of dry air and vapour in the air heating tube or rubber tube, respectively; H_1−d_ and H_1−v_ are the enthalpies of dry air and vapour in the airway, respectively; M_0−v_ is the mass of vapour in the air heating or rubber tube; and M_1−v_ is the mass of vapour in the airway.

#### Proportional reductions of dry air and vapour during the experiment

The proportions can be defined as the ratio of △Q_d_ to Q_0−d_ and △Q_v_ to Q_0−v_ for dry air and vapour, respectively. R_d_ and R_v_ can be calculated via formulas (15) and (16), as follows:


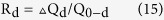



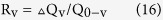


where Q_0−d_ and Q_0−v_ are the total heat quantities of dry air and vapour in the heating or rubber tube, respectively.

### Data detection and management

All data that fluctuated stably were considered valid data. The temperature and RH of the inhaled air in the trachea were recorded at 5-s intervals. The temperature varied with the breathing cycle such that temperature was higher during inspiration and lower during expiration. From these values, we chose the maximum value of temperature and its corresponding RH during each breathing cycle as inhaled air data. Furthermore, we used the HW4-E software to obtain the density of vapour (ρ_v_) and specific humidity (d) according to the temperature, RH, and atmospheric pressure. In addition, because the condition of heated air or vapour in the heating tube or rubber tube was stable, we obtained the median values of the temperature and RH to obtain constant values of H_0−d_, H_0−v_, M_d_, M_0−v_, Q_0−d,_ and Q_0−v_ across the different groups, which simplified the above calculations.

### Statistical analysis

The data are expressed as the mean ± standard deviation. Statistical analysis was performed using one-way analysis of variance (ANOVA) for comparisons of more than two groups and least squares difference (LSD-t) for comparisons of two groups (when there were differences between all groups). All statistical computations were performed using Statistical Package for the Social Sciences (SPSS) software (version 17.0; SPSS Inc., Chicago, IL, USA). The significance level was set as *P* < 0.05.

## Results

### Changes in vital signs

The oxygen saturation of all dogs was stable during the experiments, ranging between 90–100%. However, the pulse and respiratory rate were accelerated in association with the increased heat quantity of the inhaled air, especially in group IV, in which the pulse was 151.13 ± 8.00 beats per minute and the respiratory rate was 37.40 ± 2.44 breaths per minute. The breathing pattern of the dogs changed from shallow, slow breathing to deep, rapid breathing after heated air inhalation. Two dogs each in groups II, III, and IV experienced transient cough reflex and polypnea.

### Endoscopic observation

**Figure 1:**
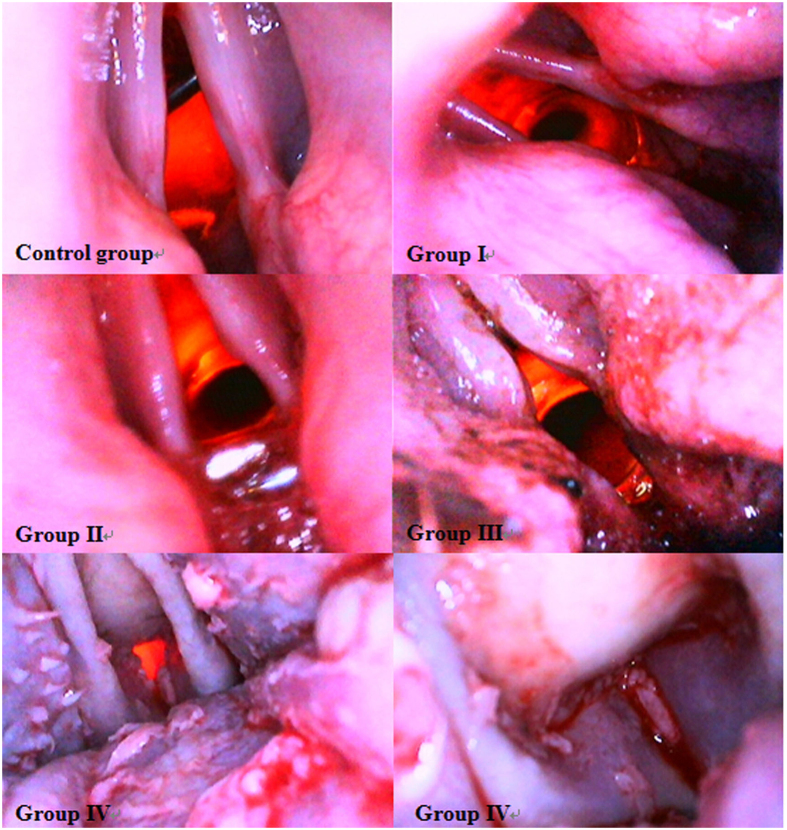
Representative images of laryngopharynxes from each of the five groups. Obvious paleness, mucosa stripping, airway stenosis, and airway obstruction are observed in group IV.

**Figure 2:**
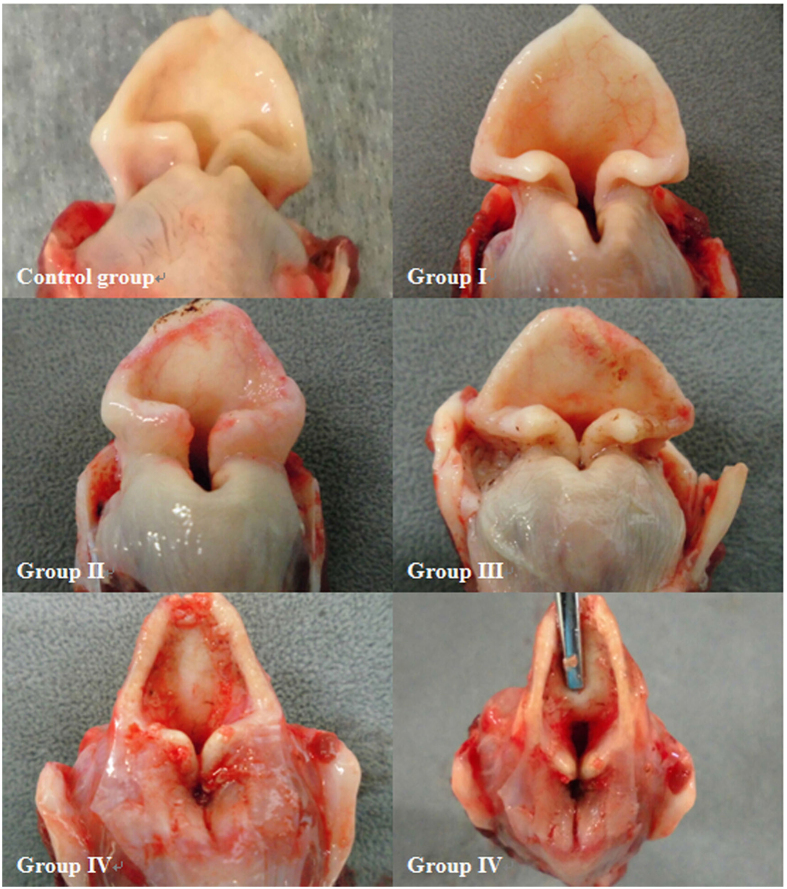
Representative images of excised laryngopharynxes from each of the five groups. Expansion and congestion of the superficial small blood vessels of the mucosa were observed in group I. Mucosal damage appeared in group II and was most evident in group IV, which exhibited severe mucosal necrosis and sloughing. In addition, obvious atrophy can be observed in group IV.

As previously established, normal laryngopharyngeal mucosa is pink, intact, and smooth without abnormal secretions. However, hyperaemic, blistering, and swollen mucosa was observed in groups I and II. Moreover, massive mucus accumulation mixed with bubbles could be found at the site of the glottis in group II. The injury in group III was more serious than that in group I and group II; more swollen and anabrotic mucosa, punctuate haemorrhage, patchy areas of paleness, and minimal mucus deposits mixed with bubbles and blood were observed. However, the most serious mucosal injury was observed in group IV, including massive oedema, extensive paleness, necrosis, sloughing, and airway obstruction (Figs 1 and 2). According to our endoscopic findings, the degree of laryngopharyngeal damage was Grade 1, Grade 1-2, Grade 3, and Grade 4 in groups I, II, III, and IV, respectively (Table 1).

### Histological findings

**Figure 3:**
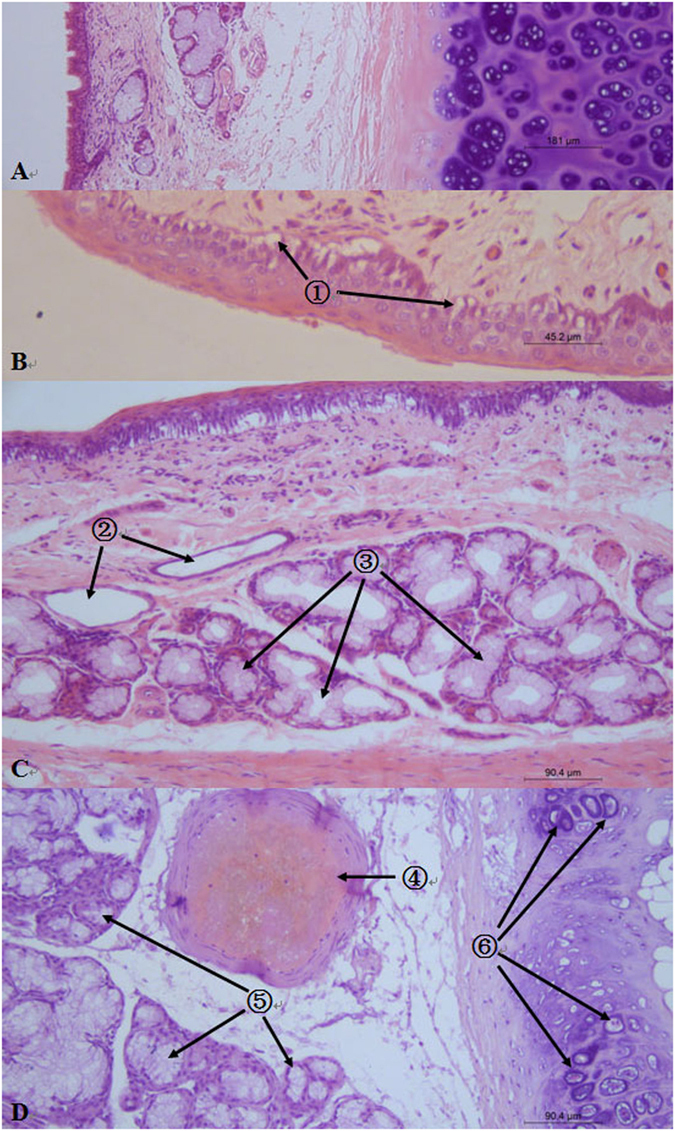
Representative images of hematoxylin and eosin-stained tissues. (**A**) Thyroid cartilage in the control group (×4 magnification). (**B**) Epiglottis in group II (×40 magnification). Symbol ① shows the swollen squamous epithelial cells. Swelling generally occurred at the bottom of the mucosa. (**C**) Epiglottis in group III (×20 magnification). Symbol ② shows the expansive blood vessels. Symbol ③ shows the swollen glands with a small glandular tube inside. (**D**) Epiglottis in group IV (×20 magnification). Symbols ④, ⑤, and ⑥ show the small thromboembolic artery, atrophic glands, and degenerative chondrocytes with eosinophilic karyopyknosis, karyorrhexis, and karyolysis, respectively.

In group I, the only histological finding was mild swelling of the squamous epithelial cells in the epiglottis. The intragroup damaging features were gradually becoming slighter from the epiglottises to cricoid cartilages when the dogs inhaled heated air of higher temperature. In group II, mild angiectasis and swollen glands in the submucosa were observed in the epiglottis, thyroid cartilage, and cricoid cartilage. In contrast, cilia exfoliation along with severe angiectasis and swollen glands was observed in group III. The most serious injury was observed in group IV, including extensive cilia exfoliation, submucosal thrombosis, glandular atrophy, and chondrocyte degeneration (Fig. 3). According to the degrees of burn severity and depth (Table 2), the degree of laryngopharyngeal damage met the criteria for first-, second-, third-, and fourth-degree damage in groups I, II, III, and IV, respectively.

### Calculation of R_d_ and R_v_

Cooling must occur when heated air enters the airway. The heat quantity of the vapour in the trachea (6.53 ± 0.28 kJ) was larger than that in the heating tube (5.82 kJ) in group I. The mass of vapour was 2.54 ± 0.11 g in the trachea and 2.20 g in the heating tube (Table 3). However, the heat quantity of dry air in the trachea was lower than that in the heating tube in all groups except for the control group.

**Figure 4:**
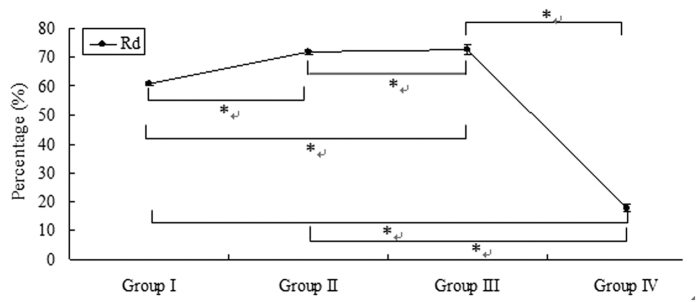
R_d_ in the different groups (%). When the dogs inhaled heated air, the increased temperature of the air did not create significant changes in R_d,_ except in group IV, where the value for the mass of dry air was significantly lower compared with the other groups. *P < 0.001.

**Figure 5:**
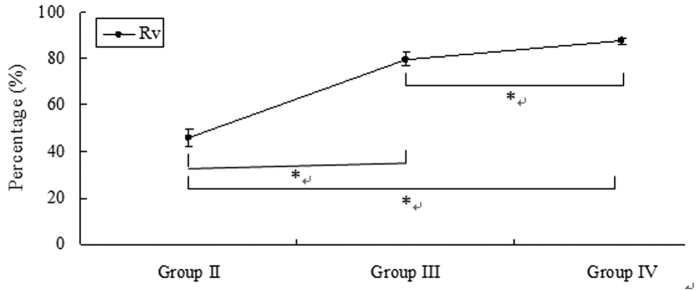
R_v_ in the different groups (%). The value of R_v_ increased as the temperature increased and the mass of vapour of inhaled heated air increased. *P < 0.001.

The temperature and RH in groups II, III, and IV were significantly different from those in the control group and group I (45.39 ± 1.00 °C and 80.03 ± 3.16% in group II, 59.44 ± 4.22 °C and 57.17 ± 6.16% in group III, and 78.80 ± 1.32 °C and 100% in group IV, respectively, *P* < 0.001). The heat quantity of dry air and vapour in the heating tube in groups II, III, and IV (9.69 kJ and 14.88 kJ; 11.24 kJ and 52.24 kJ; and 1.39 kJ and 154.61 kJ, respectively) were changed to 2.72 ± 0.06 kJ and 8.04 ± 0.52 kJ, 3.06 ± 0.21 kJ and 10.59 ± 1.46 kJ, and 1.14 ± 0.02 kJ and 19.18 ± 1.84 kJ, respectively, in the trachea. The R_d_ and R_v_ in group II were 71.89 ± 0.62% and 45.95 ± 3.47%; the corresponding values in groups III and IV were 72.73 ± 1.93% and 79.74 ± 2.80% and 17.78 ± 1.38% and 87.59 ± 1.19%, respectively (Figs 4 and 5).

## Discussion

Although the formula Q = c•m•△T may come to mind when calculating the heat quantity of materials, this formula is not suitable for calculating the heat quantity of heated air, as described in our previous study^17^. In the given formula, “c” denotes the specific heat, which depends on variables such as the number of degrees of freedom, physical state, molar mass, interior molecular forces, crystallinity, and temperature^18^. The specific heat of heated air is not constant, and instead changes with temperature and humidity. Second, the given formula requires that there is no phase change in the substance^13^,^14^. However, a decrease in temperature and increase in RH certainly occur in the airway when heated air is inhaled, indicating that the status (or phase) of heated air is thus changed.

When the dogs inhaled ordinary air, the mean temperature and RH in the trachea (37.99 ± 0.15 °C and 98.47 ± 0.58%, respectively) were both higher than those in the room (26.28 ± 1.22 °C and 54.24 ± 5.68%), indicating warming and humidification in the trachea. However, when the temperature of the inhaled air was higher than that in the trachea, cooling must have occurred.

First, if the temperature of inhaled heated air was not high enough, as in groups I and II, the total heat quantity in the heating tube and heat quantity changes between the heating tube and the trachea of dry air (Q_0−d_; △Q_d_) and vapour (Q_0−v_; △Q_v_) were nearly equal. Thermal injury in this case depended on the combined heat quantity of dry air and vapour. However, in group III, the sufficiently high temperature of inhaled heated air resulted in remarkable differences between Q_0−d_ and Q_0−v_ or △Q_d_ and △Q_v,_ suggesting that the heat quantity of moist air and its heat quantity reduction mainly depend on the vapour. Furthermore, from the increasing trends of Q_0-d_ and Q_0−v_ or △Q_d_ and △Q_v_, we can speculate that as the temperature of inhaled heated air is increased, its total heat quantity and heat quantity reduction become increasingly dependent on vapour. In other words, the thermal injury of heated air will be increasingly dependent on the heat quantity of vapour. Therefore, the heat quantity of dry air is not the main determinant of the heat quantity of moist air unless its temperature is low; thermal damage is limited in such cases.

Second, the mass of vapour in the heating tube (M_0−v_, 5.30 g in group II and 18.02 g in group III) was observed to be higher than that in the trachea (3.11 ± 0.20 g in group II and 4.05 ± 0.55 g in group III), demonstrating that vapour condensation occurred in the trachea. When evaporation is simultaneous with condensation in the same space, the dominant process will inhibit the other process^13^,^14^. From this point of thermodynamics and our experimental results, we can conclude that vapour condensation is the main process occurring in the airway rather than mucosal fluid evaporation. In other words, mucosal fluid evaporation was inhibited by vapour condensation and did not have the ability to dissipate the heat quantity of thermal air. Although mucosal fluid evaporates into vapour, it does not dissipate heat, because the vapour contains the absorbed heat and brings it back into the thermal air. Therefore, mucosal fluid evaporation is not a method of heat dissipation from thermal air inhalation injury in the airways. We propose that only heat exchange is occurring between the mucosal fluid and thermal air, which increases the temperature of the mucosal fluid but results in insufficient evaporation of the fluid in the airways and, paradoxically, inhibits vapour condensation. In addition, the most important effect of mucosal fluid is separating the mucosa from the heated air by acting as a thin protective layer. Therefore, increased mucus secretion is helpful to protect the airways in a fire.

Third, an important reason for vapour condensation is that convection occurs between the inhaled heated air and cold air in the trachea during every breath^19^. Hence, to cool and exhale the heated air as soon as possible, or due to stimulation by the heated air, the breathing patterns of each dog were altered from shallow, slow breathing to deep, rapid breathing after heated air inhalation. However, in an actual fire, we cannot and should not quicken or deepen our breath; instead, we should hold our breath, decrease its frequency, reduce the speed of inspiration, and quicken expiration to decrease the heat forced into the airway.

Fourth, when the dogs inhaled saturated vapour in group IV, the RH was 100% at all times, both in the rubber tube and trachea. Mucosal fluid evaporation could not occur, and vapour condensation was noted in the trachea only. Based on the law of the conservation of energy^13^,^14^, where did the △Q_v_ go? It is possible that the decreased heat quantity was dissipated by the blood circulation and accepted by the airway tissue^17^. The airway tissue here refers mainly to the mucus adhering to the mucosa, the mucosa itself, and the submucosal soft tissue. Further explorations on circulational heat dissipation and airway tissue heat acceptance (especially in the mucus and mucosa) will help us better understand thermal inhalation injury.

The anatomic site of the laryngopharynx positions it directly in the path of inhalants of all types as they flow from the mouth or nose to the lungs. The laryngopharynx is an irregular tubular structure and is the narrowest part of the upper air tract^20^, making it particularly vulnerable to severe injury^21^. In the present study, we found obvious damage to the laryngopharyngeal mucosa by macroscopic and histological observations. In particular, group IV exhibited fourth-degree burns, which are fatal to dogs. One direct reason for this observed airway tissue damage is the high total heat quantity of inhaled air. To dissipate the heat as quickly as possible, all dogs deepened and accelerated their respiration, which resulted in increasingly serious damage to the airway tissue. This vicious cycle may play an important role in the observed damage.

Another reason for observed airway tissue damage is the heat quantity of vapour in inhaled heated air. Moist air has more heat to dissipate than an equal volume of dry air at any temperature^22^, given that the vapour in moist air has much higher heat-carrying capacity than that in dry air^23^,^24^. Vapour has a heat-carrying capacity 4,000 times that of ordinary heated air. Hence, more severe pulmonary injuries may be encountered from inhalation of vapour than from smoke or dry air^23^,^24^. In the present study, as mentioned above, △Q_v_ and R_v_ increased as the temperature of the inhaled air increased. In particular, △Q_v_ and R_v_ were 135.43 ± 1.84 kJ and 87.59 ± 1.19%, respectively, and the heat quantity of the inhaled vapour was 111.23 times that of dry air in group IV. Thus, the tremendous heat quantity of vapour released in the airway resulted in the most serious full-thickness airway damage in group IV compared to the other groups, despite the circulational heat dissipation in this group^17^. Furthermore, we speculate that the airway tissue damage will not be serious if there is no vapor, even if the temperature of the inhaled air is high enough, owing to the low heat capacity and seemingly constant △Qd and Rd, indicating that the surrounding air humidity also plays a key role in determining the extent of thermal injury.

Vapour inhalation is often advised as therapy for upper airway infections. However, serious complications such as vapour burns may occur, especially in children^25^,^26^. Although we did not examine the lower airway in group IV, we can deduce that lower airway tissue damage is likely to be serious given that only vapour is capable of inflicting direct thermal damage to the lower airways. Therefore, some researchers have advised that vapour inhalation therapy should no longer be recommended^22^.

There were some limitations to our study. First, we used the tidal volume of dogs in our calculations. As mentioned above, all dogs displayed deeper respiration after inhaling heated air, which resulted in calculated values for mass and heat quantity of dry air and vapour that were lower than the actual values. Second, because of the continuous breathing of the dogs, the water droplets promptly covered the surface of the lens when our endoscope entered into the deep oral cavity. There also was a considerable amount of light reflected from the mucosa. All these factors lowered the resolution of our images. Third, the nasal cavity is an important upper airway structure. However, we did not investigate laryngopharyngeal injury in cases where the nasal cavity was utilized during the breathing of thermal air.

## Additional Information

**How to cite this article**: Wan, J.-b. *et al*. Mucosal fluid evaporation is not the method of heat dissipation from fourth-degree laryngopharyngeal burns. *Sci. Rep.* **6**, 28772; doi: 10.1038/srep28772 (2016).

## Supplementary Material

Supplementary Information

## Figures and Tables

**Table 1:** Grading of laryngopharyngeal mucosal damage on endoscopy.

**Grade**	**Finding**
Grade 0	No injury (absence of hyperemia, edema, blistering)
Grade 1	Minimal hyperemia, edema, and/or blistering, with or without mucus deposit
Grade 2	Moderate hyperemia, edema, and/or blistering, with or without mucus deposit
Grade 3	Severe hyperemia, edema, or patchy areas of paleness, anabrosis, punctuate hemorrhage, with or without mucus deposit
Grade 4	Massive mucosal hyperemia, edema or extensive mucosal paleness, necrosis, sloughing, punctuate hemorrhage, with airway obstruction, with or without mucus deposit

**Table 2:** Histological degrees of laryngopharyngeal burns.

**Pathological changes**	**Degree**			
	**First**	**Second**	**Third**	**Fourth**
Mucosa				
Cilia exfoliation^#^	None	None	<1/2	≥1/2
Epithelial cells	Mild swelling	Moderate swelling	Severe swelling, disorganised^$^	Severe swelling, disorganised^$^
Submucosa				
Blood vessels	Normal	Mild angiectasis	Severe angiectasis	Thrombosis
Glands	Normal	Mild swelling	Severe swelling	Atrophy
Chondrocytes	Normal	Normal	Normal	Degeneration

^#^Length proportion of exfoliated cilia, as observed in a 20× field.

^$^Residual epithelial cells.

**Table 3:** The mass and heat quantity of dry air and vapour.

	**The mass in the heating or rubber tube (g)**		**The mass in the trachea (g)**		**The heat quantity in the heating or rubber tube (kJ)**		**The heat quantity in the trachea (kJ)**	
	Dry air	Vapour	Dry air	Vapour	Dry air	Vapour	Dry air	Vapour
Control group	52.76	0.61	52.76	2.26 ± 0.34	1.39	1.56	2.01 ± 0.01	5.81 ± 0.01
Group I	56.01	2.20	56.01	2.54 ± 0.11	5.65	5.82	2.22 ± 0.04	6.53 ± 0.28
Group II	59.70	5.30	59.70	3.11 ± 0.20	9.69	14.88	2.72 ± 0.06	8.04 ± 0.52
Group III	51.31	18.02	51.31	4.05 ± 0.55	11.24	52.24	3.06 ± 0.22	10.59 ± 1.46
Group IV	14.43	57.74	14.43	7.25 ± 0.69	1.39	154.61	1.14 ± 0.02	19.18 ± 1.84
