# Supplementary material for: Mucosal fluid evaporation is not the method of heat dissipation from fourth-degree laryngopharyngeal burns
Source: Sci Rep. 2016 Jun 28;6:28772. doi: 10.1038/srep28772 (PMC4923872; doi:10.1038/srep28772)
Supplement: Supplementary Information [file srep28772-s1.doc]

**Mucosal fluid evaporation is not the method of heat dissipation from fourth-degree laryngopharyngeal burns**

Jiang-bo Wan^1^, Guo-an Zhang^1^, Yu-xuan Qiu^1^, Chun-quan Wen^1^, Tai-ran Fu^2^

^1^ Department of Burns Surgery, Peking University Fourth School of Clinical Medicine, No. 31, Xinjiekou East Street, Xicheng District, Beijing 100035, PR China.

^2^ Key Laboratory for Thermal Science and Power Engineering of Ministry of Education, Department of Thermal Engineering, Tsinghua University, No. 1, Tsinghua Park, Haidian District, Beijing 100084, PR China.

* Corresponding author. E-mail: daoshizga@163.com; Tel.: +86 010 58516361; Fax: +86 010 58516719.


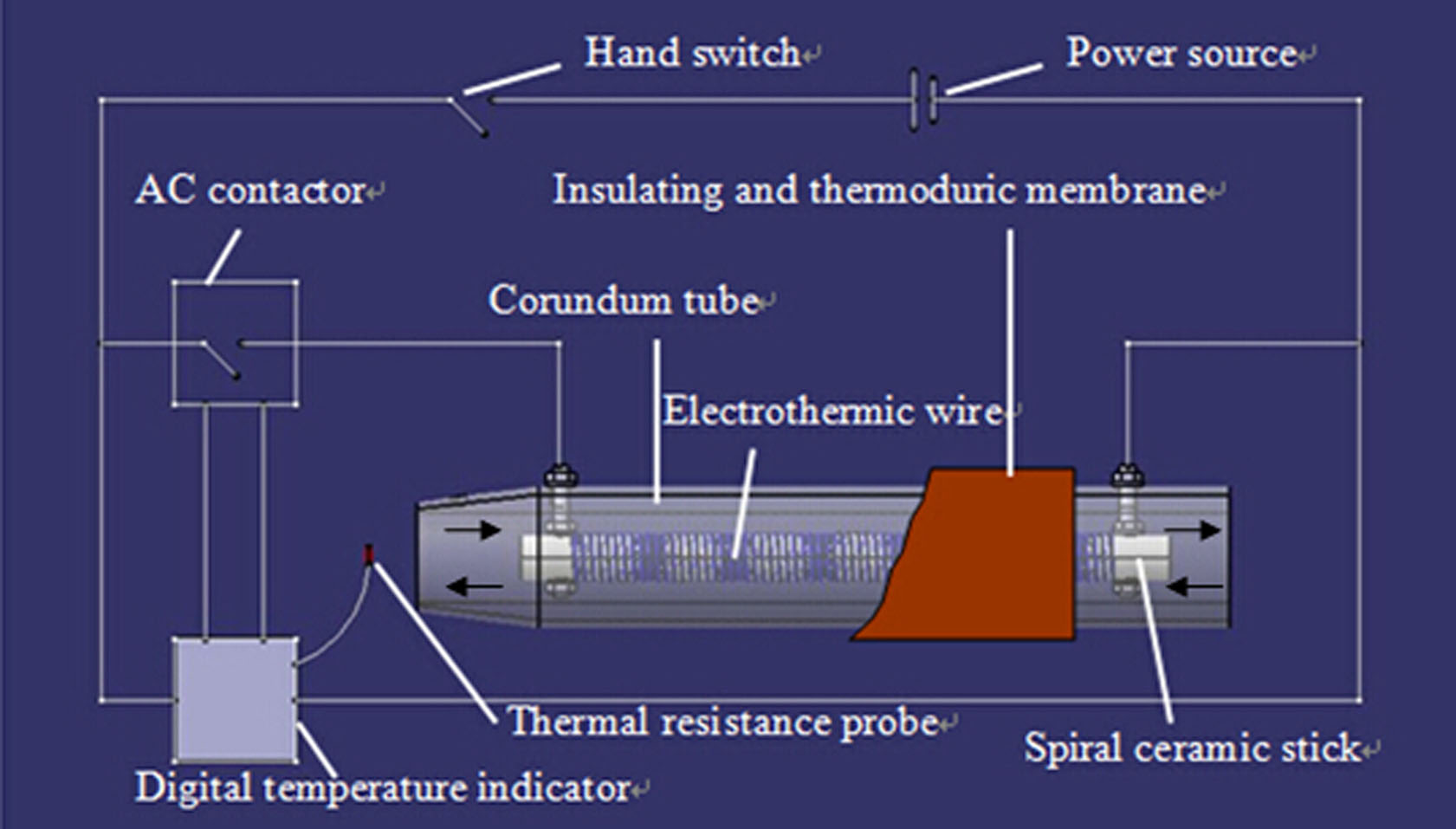


Fig. 6. Experimental air heating tube. Arrows show the direction of gas flow. If the dogs inhaled heated air, then a thermal resistance probe was secured in the deep oral cavity with sutures, with its end approximately 1 cm in front of the heating tube without tissue contact.


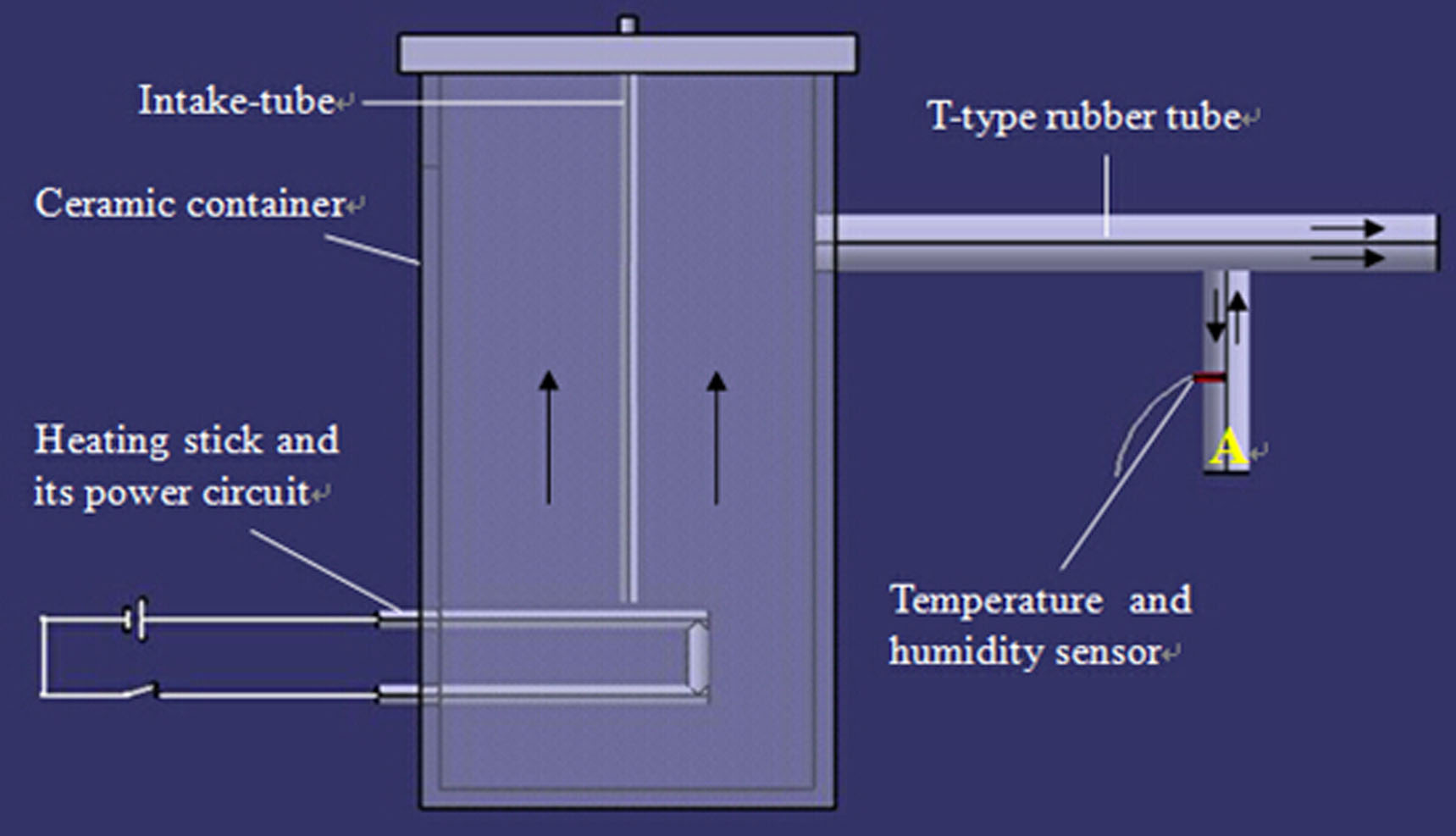


Fig. 7. Experimental vapour generator. Arrows show the direction of gas flow. If the dogs inhaled saturated vapour, then the end of the vertical axis (position A) was placed in the deep oral cavity. The end of the temperature and humidity sensor was fixed inside the deep oral cavity without tissue contact. Meanwhile, oxygen (5 l/min) was supplied through a built-in stainless intake tube.
